# α-Latrotoxin and Pain: A Nociceptor Perspective on Latrodectism

**DOI:** 10.3390/toxins18090393

**Published:** 2026-09-10

**Authors:** Bazbek Davletov, Alexandr Ignatchenko, Aisha Zhantleuova, Rashid A. Giniatullin

**Affiliations:** 1 Department of Biophysics, Biomedicine and Neuroscience, Farabi University, Almaty A15E3C7, Kazakhstan; aisha.zhantleuova@kcl.ac.uk; 2 Department of Biomedical Science, University of Sheffield, Sheffield S10 2TN, UK; 3 European Molecular Biology Laboratory, European Bioinformatics Institute, Hinxton CB10 1SD, UK; alexsign@ebi.ac.uk; 4 Wolfson Centre for Age-Related Diseases, Institute of Psychiatry, Psychology and Neuroscience, King’s College London, London SE1 1UL, UK; 5 A.I. Virtanen Institute for Molecular Sciences, University of Eastern Finland, 80101 Kuopio, Finland; rashid.giniatullin@uef.fi

**Keywords:** alpha-latrotoxin, nociception, nociceptor, dorsal root ganglion, pore-forming toxin

## Abstract

Alpha-latrotoxin (α-LTX) is the principal vertebrate-specific neurotoxin in widow spider (*Latrodectus*) venom and a powerful presynaptic secretagogue. Although its receptors, pore-forming activity and stimulation of neurotransmitter release have been studied extensively in central and motor neurons, its relationship to the severe, persistent pain of latrodectism remains poorly understood. This focused review re-examines α-LTX from a nociceptive perspective. The available evidence supports a model in which α-LTX binds adhesion G protein-coupled receptor L1 (ADGRL1; latrophilin-1) and neurexin-1α on susceptible sensory neurons; inserts a large cation-permeable pore; and promotes membrane depolarisation, calcium entry and release of pain-associated neuropeptides. The transcript-level gene expression of α-LTX receptor genes in dorsal root ganglion neurons and evidence of toxin-evoked neuropeptide release provide a molecular basis for direct nociceptor activation, although functional validation in defined nociceptor subclasses remains necessary. A comparison with the nociceptive ion channels transient receptor potential vanilloid 1 (TRPV1) and transient receptor potential ankyrin 1 (TRPA1) highlights their convergence on calcium-dependent sensory excitation, while distinguishing α-LTX from toxins that modulate endogenous channels. We propose that the pain of latrodectism is a composite state in which direct nociceptor activation complements muscle spasm and tissue-derived signalling, positioning α-LTX as a distinctive probe of pain pathways.

## 1. Clinical Pain in Latrodectism

**Figure 1:**
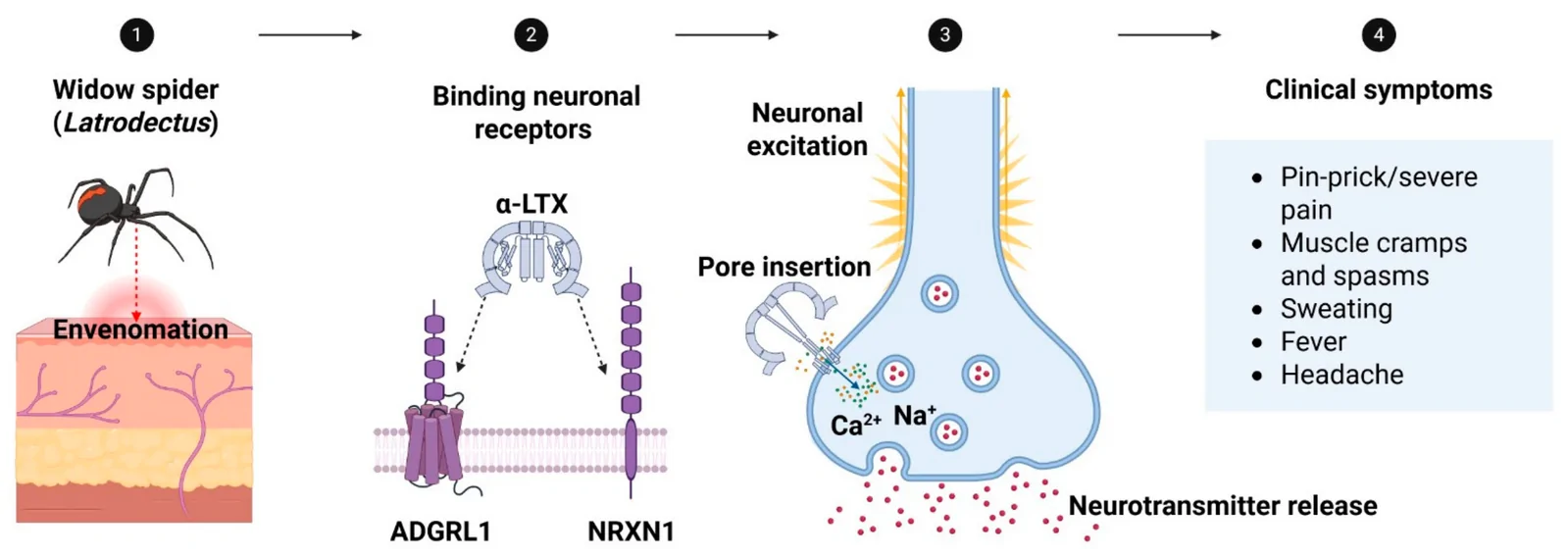
Illustrative pathway showing widow spider envenomation, leading to α-latrotoxin binding to neuronal receptors and pore formation, triggering the influx of ions into nerve cells, particularly Ca^2+^, neuronal excitation and multiple biological effects. Created in BioRender. Zhantleuova, A. (2026), https://BioRender.com/5v9yl89.

Widow spider bites typically occur when humans come into direct contact with their webs. When bitten (Figure 1), the venom causes latrodectism, with symptoms such as a sharp, pinprick-like pain; muscle cramps and spasms that start near the bite and then spread through the body; along with sweating, fever, and headache [1,2]. The management of mild-to-moderate latrodectism is primarily supportive, while severe cases are treated with intravenous calcium, muscle relaxants, widow spider antivenom and analgesic opioids [3]. Most people recover significantly within three to four days, with the worst symptoms occurring during the first 24 h [2,4]. Latrodectism remains a global health challenge, especially in developing countries [1,3]. Although the venoms of spiders of the genus *Latrodectus* contain various components, α-latrotoxin, a large multidomain protein, is the principal toxin involved in vertebrate latrodectism [5].

Neurotoxins have evolved to manipulate the nervous system with remarkable precision. Their actions can produce markedly different functional outcomes [6,7]. At one end of this spectrum, some animal venom toxins, e.g., α-bungarotoxin from snake venom, suppress neuromuscular transmission and promote immobilisation [8]. At the other end, some tarantula venom components appear to have evolved to produce a rapid and intense activation of nociceptive pathways, thereby generating pain that can serve as a powerful defensive signal [9,10,11]. The resulting rapid excitation of nociceptors can elicit an immediate behavioural response capable of deterring a predator without requiring lethality. Importantly, these functional outcomes are not mutually exclusive. Many animal venoms produce both pain and motor hyperexcitation, including muscle spasms and spastic paralysis. Scorpion venoms provide a prominent example, as their neurotoxins can simultaneously evoke intense pain through actions on nociceptive pathways and produce motor hyperexcitability associated with muscle spasms and paralysis [8]. Thus, individual toxins may contribute to both defensive and predatory functions, and their paralytic and nociceptive effects should be viewed as potentially overlapping rather than strictly opposing neurobiological strategies.

Severe pain in latrodectism has traditionally been largely attributed to sustained muscle contractions caused by α-LTX. Muscle spasms undoubtedly contribute to the clinical syndrome, but several features suggest the additional involvement of more direct nociceptive mechanisms. These include pain or burning at the bite site, local or regional hyperalgesia, and erythema appearing as a red ring immediately surrounding the two tiny puncture marks [1,2]. These clinical observations raise three fundamental questions:

- To what extent is pain in latrodectism independent of muscle spasm?
- Are sensory neuron populations responsive to α-LTX?
- Does α-LTX directly activate peripheral nociceptors?

Surprisingly, these questions remain poorly investigated.

Disorders in which muscle spasm is the principal pathological process provide a useful, although imperfect, comparison. In tetanus, for example, tetanus neurotoxin blocks transmitter release from inhibitory interneurons, resulting in motor neuron disinhibition, rigidity, and painful muscle spasms [6,12]. Pain is therefore generated largely as a downstream consequence of motor system dysfunction. Conceptually, this can be distinguished from nociceptor-driven pain arising through the primary excitation of sensory neurons. Spasm-associated pain develops through sustained contraction, local ischaemia, mechanical stress, and the accumulation of algogenic metabolites, which subsequently activate muscle afferents [7,13]. Direct nociceptor excitation, by contrast, can produce rapid, intense afferent activity before or independently of substantial muscle contraction [7,13]. These mechanisms are not mutually exclusive and may theoretically reinforce one another during latrodectism.

It is conceivable that α-LTX acts at the intersection of these two mechanisms. Activation of motor pathways produces painful muscle contraction, while the potential expression of α-LTX receptors by sensory neurons would allow α-LTX to also target nociceptors. Latrodectism may therefore represent a composite pain state in which primary sensory neuron excitation is amplified by secondary muscle spasm and tissue-derived nociceptive signals. From this perspective, α-LTX could be a candidate pain-producing toxin whose receptor engagement, pore formation, calcium signalling, and stimulation of neurosecretion may provide a distinctive framework for investigating the molecular initiation and persistence of pain.

## 2. Molecular Targets of Alpha-Latrotoxin and Pore Formation

Remarkably, despite pain being the defining clinical feature of latrodectism [3], mechanistic studies of α-LTX have overwhelmingly focused on neurotransmitter release at motor and central synapses rather than on nociceptive neurons [14]. Early investigations naturally focused on motor and central neurons for several reasons. First, the quantifiable nature of muscle spasms and the large size of the neuromuscular junctions were beneficial for initial studies, allowing for the direct visualisation and quantitative analysis of secretory vesicle depletion following α-LTX exposure [15]. Second, α-LTX was used to robustly stimulate neurotransmitter release from numerous brain preparations, providing abundant material for biochemical purification and characterisation of its receptors [14,16]. Third, at the time these studies were initiated, the molecular diversity of sensory neurons and the existence of genetically defined nociceptor subtypes were largely unknown [17,18]. Consequently, there was little conceptual framework for investigating selective actions of α-LTX on nociceptive circuits. The experimental advantages of motor and central neurons drove many of the major advances in understanding neurosecretion and led to the identification of the principal neuronal receptors for α-LTX [16,19,20,21]. Today, however, advances in the molecular biology of sensory neurons have revealed the remarkable molecular and functional diversity of nociceptors [18], providing an opportunity to re-examine the actions of α-LTX through the lens of nociceptive biology.

Receptor-targeted neurotoxins can define and control neuronal circuits by selectively engaging distinct neuronal populations. α-LTX engages neurons via receptor interactions by binding latrophilin-1 and neurexin-1α, key components of synaptic architecture, triggering massive neurotransmitter release across synapses [16,20,21]. Latrophilin-1 was initially given an additional name—CIRL1, for calcium-independent receptor for latrotoxin 1 [20]. However, following revision of the adhesion GPCR nomenclature [22], it was renamed ADGRL1 (adhesion G protein-coupled receptor L1). Importantly, the family designation “L” retains the historical connection to latrophilin, reflecting its original identification as the major high-affinity receptor for α-LTX. Additional binding partners, including the receptor-type protein tyrosine phosphatase PTPσ, have been reported, although their physiological significance remains considerably less well established than that of ADGRL1 and neurexin-1α [23]. Therefore, further discussion will focus on the two principal neuronal receptors for α-LTX: ADGRL1 (latrophilin-1) and neurexin-1α, which together account for most mechanistic studies on toxin binding and action.

**Figure 2:**
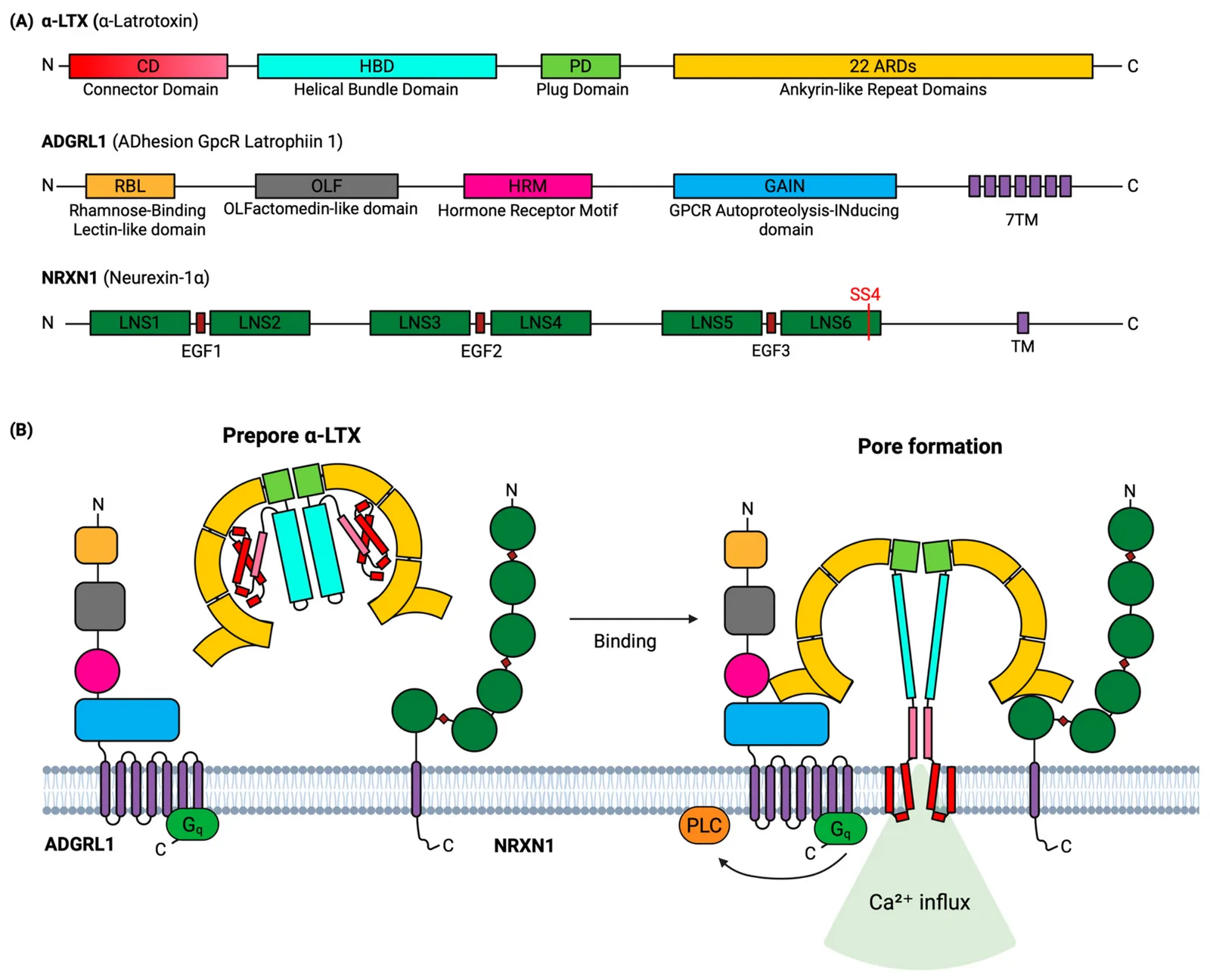
Schematic representation of α-LTX structure, its principal receptors, and the mechanism of pore formation. (**A**) Schematic domain organisation of α-LTX (**top**), the adhesion G protein-coupled receptor ADGRL1/latrophilin-1 (**middle**), and neurexin-1α (**bottom**). TM, transmembrane domain; LNS, laminin–neurexin–sex hormone-binding globulin domain; EGF, epidermal growth factor-like domain; SS4, alternative splice site 4. Alternative splicing at SS4 (red) strongly influences the ability of neurexin-1α to bind and support the activity of α-LTX. Schematics are not drawn to scale. (**B**) α-LTX exists as tetramers in the presence of extracellular calcium (for clarity, only two opposing α-LTX monomers are depicted in the two-dimensional schematic). Following its receptors’ binding, the α-LTX tetramer undergoes conformational rearrangement, forming a Ca^2+^-permeable transmembrane pore. Schematics are adapted from published structural receptor models [5,32,33]. Colours are used to indicate the same structural domains across the panels. Created in BioRender. Zhantleuova, A. (2026), https://BioRender.com/nwfms0w.

ADGRL1 and neurexin-1α naturally evolved as endogenous neuronal proteins with essential roles in synapse formation, organisation, and function (Figure 2). Although structurally unrelated, both participate in trans-synaptic adhesion, thereby contributing to the establishment and maintenance of neuronal connectivity. ADGRL1 is a ~180 kDa adhesion G protein-coupled receptor (adhesion GPCR) expressed predominantly in neurons. Like other members of this receptor family, it combines adhesive extracellular domains with a seven-transmembrane GPCR architecture, enabling it to function both as a cell-binding molecule and as a signalling receptor [22]. Its large extracellular region contains multiple protein interaction domains that mediate trans-synaptic binding, whereas the intracellular C-terminal region can couple to heterotrimeric G proteins and downstream signalling pathways, such as phospholipase C. The extracellular domain of ADGRL1 comprises a rhamnose-binding lectin-like (RBL) domain, an olfactomedin-like domain, a hormone receptor motif (HRM), and the GPCR Autoproteolysis-INducing (GAIN) domain containing the GPCR proteolysis site (GPS) [24]. Autoproteolytic cleavage at the GPS generates an N-terminal fragment (NTF) and a C-terminal fragment (CTF), which remain non-covalently associated at the cell surface. The large extracellular region of ADGRL1 binds teneurins/Lasso and other ligands [25,26], but can also serve as the binding platform for α-LTX, allowing the toxin to exploit an endogenous synaptic adhesion receptor for selective targeting of neuronal terminals.

Neurexin-1α is also a large presynaptic cell adhesion protein; it belongs to the extensive neurexin family [27]. Unlike ADGRL1, neurexin-1α is not a GPCR but a single-pass transmembrane glycoprotein whose extracellular domain organises trans-synaptic contacts through interactions with several postsynaptic ligands, such as neuroligins, leucine-rich repeat transmembrane proteins (LRRTMs), and secreted bridging molecules such as cerebellins [28]. Extensive processing with alternative splicing generates hundreds of neurexin isoforms, providing remarkable molecular diversity across neuronal populations for endogenous adhesion interactions [27,29]. Alternative splicing regulates the ability of neurexins to bind their trans-synaptic partners, but also to function as α-LTX receptors. Splicing at site 4 (SS4), located within the sixth laminin–neurexin–sex hormone (LNS) domain, strongly influences toxin binding and receptor activity, with inclusion of the SS4 insert markedly reducing the responsiveness of the relevant neurexin variants to α-LTX [30]. Thus, α-LTX does not recognise neurexins indiscriminately but distinguishes between structurally defined splice variants. This work provided an early example of alternative splicing controlling the activity of a neuronal toxin receptor and, consequently, cellular susceptibility to a neurotoxin [30,31]. More broadly, the ability of α-LTX to exploit two structurally unrelated organisers of synaptic architecture—ADGRL1 and neurexins—is a striking feature of its neuronal specificity.

Despite their structural differences, under physiological conditions ADGRL1 and neurexins can similarly recruit α-LTX to the neuronal surface and support its pore-forming activity [34,35]. Once inserted, the pore functions independently; the receptors merely act as tethers to facilitate insertion rather than forming part of the channel structure itself [34,35]. Unlike toxins that modify endogenous ion channels, α-LTX is itself a large multidomain pore-forming protein [5,32]. Each toxin monomer comprises an N-terminal connector domain, a central helical-bundle domain, a small β-sheet plug domain, and a C-terminal region containing 22 ankyrin repeats arranged into a curved solenoid (Figure 2) [32]. Importantly, at a physiological extracellular Ca^2+^ concentration, α-LTX exists predominantly as a tetramer, in which the ankyrin-repeat shell shields the hydrophobic membrane-inserting machinery [5,32,36]. We propose that in the case of tetrameric α-LTX, simultaneous engagement of several ADGRL1 and/or neurexin-1α molecules by the ankyrin-repeat shell could induce stretching of the shell, resulting in conformational rearrangements that would expose the α-LTX membrane-inserting machinery. Recent cryo-electron microscopy studies resolved both prepore and pore conformations of the α-LTX tetramer [32]. Transition to the pore state involves extensive rearrangement of the N-terminal domains. The four helical bundles refold to form an approximately 15 nm coiled-coil stalk that inserts the N-terminal membrane-spanning helices into the lipid bilayer, thereby creating a cation-permeable channel. The large non-selective cation-conducting pore allows a massive Na^+^ and Ca^2+^ influx, leading to excitation and catastrophic neurotransmitter release [37]. In this way, receptor binding determines where α-LTX acts, whereas pore formation determines how it acts. It is worth mentioning that the enormous ankyrin-repeat scaffold distinguishes α-LTX from virtually all other pore-forming neurotoxins and suggests an intriguing parallel with ankyrin-containing nociceptive ion channels, particularly TRPA1 and TRPV1 [32]. The possible functional significance of this parallel is considered later in this review.

## 3. Do Nociceptors Express α-LTX Receptors?

Single-cell and single-nucleus transcriptomics have greatly advanced our understanding of sensory neuron diversity by identifying molecularly distinct populations, including multiple classes of nociceptors [18]. These approaches now make it possible to address a fundamental question in α-LTX biology: do nociceptors express the neuronal receptors necessary for toxin binding and subsequent membrane pore insertion? When the molecular mechanisms of α-LTX were originally established, this question could not be addressed because neither the molecular markers nor the transcriptomic methods [18] needed to resolve nociceptor populations were available.

Evidence connecting ADGRL1 to sensory function was initially obtained in Drosophila. The fly ADGRL1 orthologue CIRL is expressed in both low-threshold mechanoreceptors and high-threshold nociceptors, in which it exerts opposing effects on mechanosensory processing [38]. CIRL enhances responses to innocuous mechanical stimuli while attenuating nociceptor responses to noxious mechanical stimulation, thereby improving discrimination between gentle and harmful touch. These findings establish an endogenous role for the ADGRL1 family in sensory processing and pain-related behaviour. However, because α-LTX is vertebrate-specific, the Drosophila results do not demonstrate that fly CIRL functions as an α-LTX receptor [38].

The same study examined latrophilin expression in rat dorsal root ganglia and detected ADGRL1 and its isoform ADGRL3 transcripts in several classes of primary sensory neurons. These included CGRP-positive peptidergic nociceptors, IB4-binding non-peptidergic nociceptors, and NF200-positive myelinated sensory neurons. Following chronic constriction injury, ADGRL1 expression was transiently reduced in IB4-positive neurons. These observations demonstrate that ADGRL1 is not restricted to central or motor neurons but is also expressed by mammalian primary sensory neurons, including major classes of nociceptors. They also suggest that its expression may be dynamically regulated after peripheral nerve injury [38].

Transcriptomic studies have provided further evidence that the two principal α-LTX receptor genes are expressed in vertebrate sensory ganglia. A pharmacological target-focused analysis of native human and mouse DRG detected ADGRL1 among the GPCR transcripts expressed in primary sensory tissue [39]. More recent human DRG atlases have identified ADGRL1 in several sensory neuron populations, including nociceptor-associated clusters, with further expression observed in low-threshold mechanoreceptors and that appeared particularly prominent in A-delta (Aδ) low-threshold mechanoreceptors. Thus, ADGRL1 is not a selective nociceptor marker but is present in neuronal populations, capable of contributing to both nociceptive and mechanosensory responses [40].

**Figure 3:**
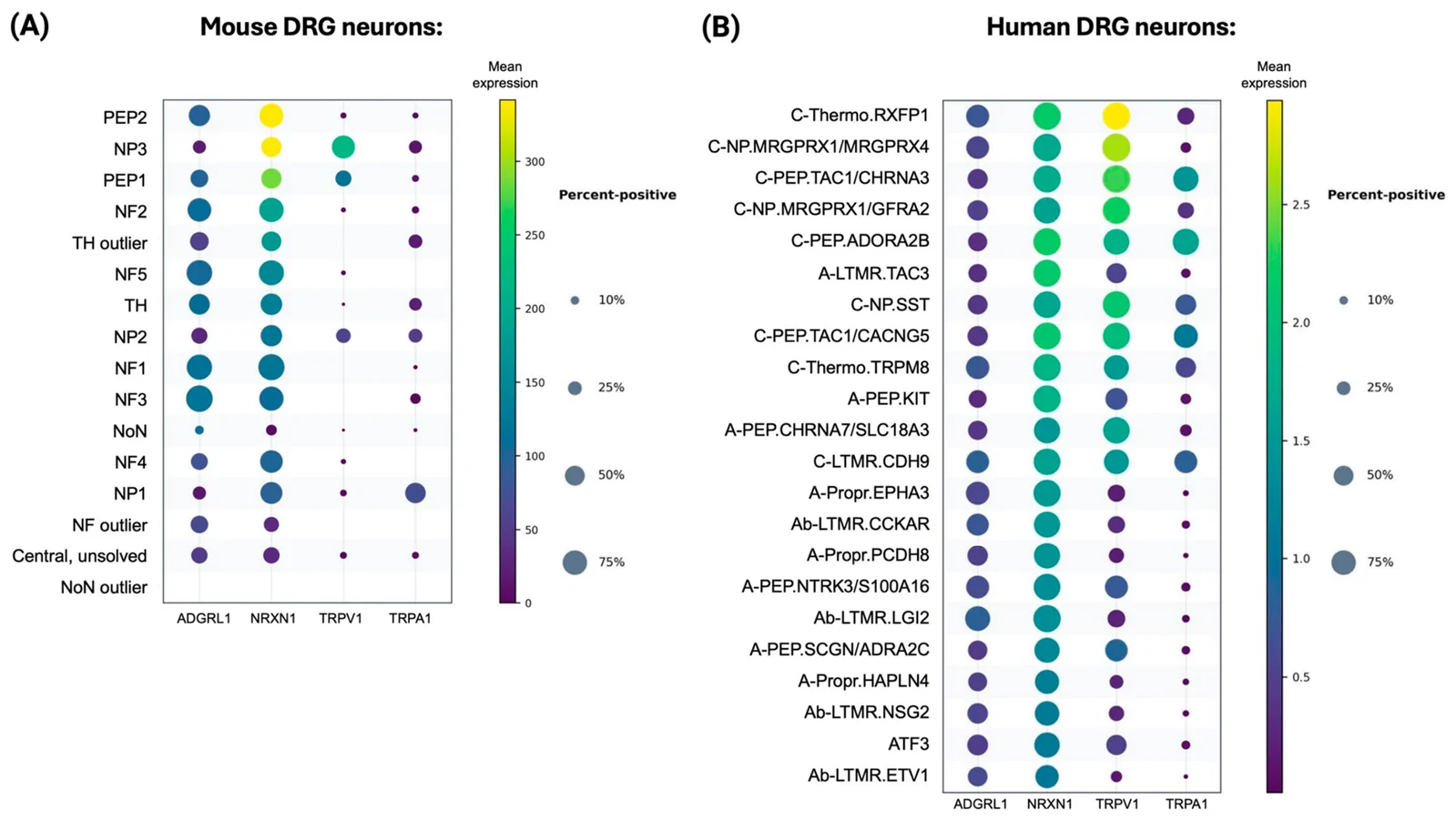
Dot plots showing transcript-level expression of ADGRL1, NRXN1, TRPV1, and TRPA1 across mouse (**A**) and human (**B**) dorsal root ganglion sensory neuron classes, based on reanalysis of public datasets [18,41]. The x-axes show genes and the y-axes show neuronal classes as presented in [18,41]. The dot size represents the percentage of cells with detectable expression, and the dot colour represents the mean expression across all cells in the class.

Our investigation of the most recent human and mouse DRG transcriptomic datasets similarly indicates that nociceptor populations, including those expressing TRPV1 and/or TRPA1, co-express the canonical α-LTX receptor genes ADGRL1 and NRXN1, albeit with different distributions (Figure 3; Tables S1–S7) [18,41]. In our analysis, we recalculate, for each annotated cell group and each gene, the total number of cells, the mean expression across all cells, the median expression, and the percentage of cells with detectable expression. Figure 3 shows that NRXN1 is detected broadly across sensory neuron populations, whereas ADGRL1 is present in a smaller but still substantial fraction of nociceptors. This overall pattern appears conserved between the human and mouse datasets analysed here.

Several limitations must nevertheless be considered. Detection of NRXN1 transcripts does not necessarily establish expression of the neurexin-1α isoform or determine the inclusion of the SS4 insert, because most single-cell and single-nucleus sequencing methods do not reliably resolve promoter usage or splice isoforms. Similarly, transcript detection does not prove that the corresponding receptor protein is present and accessible at peripheral nociceptor endings. Transcriptomic dropout may also underestimate the proportion of receptor-expressing neurons. Most importantly, receptor expression establishes susceptibility in principle but does not demonstrate that α-LTX directly binds to or activates nociceptors.

Taken together, the available evidence demonstrates that the molecular machinery required for α-LTX recognition is present in mammalian sensory neurons, including nociceptors. This provides a plausible molecular basis for direct toxin action on pain-sensing neurons and supports the possibility that latrodectism involves primary activation of sensory pathways in addition to pain generated secondarily by muscle spasm [38,39,40].

The next question is whether α-LTX has been shown experimentally to act on primary sensory neurons or their peripheral terminals. Although the available evidence is limited, several studies have indicated that α-LTX can evoke the release of neuropeptides implicated in nociception. These mediators, including tachykinins and CGRP, can contribute to neurogenic inflammation and peripheral and central sensitisation.

Waterman and Maggi directly investigated whether α-LTX could stimulate neuropeptide release from primary afferent and enteric neurons in guinea pig tissues. They found that α-LTX evoked neuropeptide-mediated responses in both sensory and enteric preparations, providing early functional evidence that the toxin can activate peptidergic sensory pathways [42]. Additional evidence came from an isolated vascular preparation, in which an α-LTX-induced smooth-muscle response was abolished by the CGRP receptor antagonist CGRP (aa 8–37). Although CGRP release was not measured directly, this pharmacological result is consistent with CGRP-dependent sensory nerve signalling [43].

More direct cellular evidence was provided by Nakata and colleagues, who visualised α-LTX-induced synaptic vesicle exocytosis in cultured adult mouse DRG neurons expressing synaptophysin–GFP. Exposure to α-LTX triggered massive exocytosis, with almost all labelled synaptic vesicles undergoing fusion with the plasma membrane [44]. This experiment established that DRG neurons possess α-LTX-responsive exocytic machinery, although it did not identify the sensory neuron subtypes involved or the cargo released [44].

Together, these findings do not yet demonstrate that α-LTX directly activates defined mammalian nociceptor populations in vivo. Nevertheless, they connect receptor transcripts in sensory neurons with functional evidence of neuropeptide signalling and strong vesicular exocytosis. Combined with its receptor specificity, pore-forming activity, depolarisation and Ca^2+^ influx [37], these properties support a model in which α-LTX directly engages with sensory pathways and induces the release of pain-associated mediators. Reframing α-LTX as a probe of nociceptive circuits may therefore reveal a previously underappreciated dimension of this extensively studied neurotoxin [38,42,43,44].

## 4. Comparison with TRPV1, TRPA1, and Pain-Producing Venom Toxins

A useful conceptual framework is to compare α-LTX with endogenous cation channels that initiate nociceptive signalling. Following receptor engagement, α-LTX undergoes membrane insertion and forms a tetrameric cation-selective pore [36]. The resulting Na^+^ and Ca^2+^ influx can depolarise neurons and stimulate transmitter release. In this functional sense, α-LTX produces an outcome resembling prolonged activation of nociceptive transient receptor potential (TRP) channels [32,34,35,45,46,47].

TRPA1 (transient receptor potential ankyrin 1) and TRPV1 (transient receptor potential vanilloid 1, historically known as the capsaicin receptor) are particularly relevant comparators. Both are tetrameric, Ca^2+^-permeable cation channels expressed by nociceptors and strongly implicated in chemically induced and inflammatory pain [45,46,47]. Both also contain cytoplasmic N-terminal ankyrin-repeat domains, although the array is considerably larger in TRPA1 than in TRPV1. These domains contribute to channel regulation, ligand-dependent modulation and interactions with cellular proteins. α-LTX similarly possesses an extensive ankyrin-repeat region, comprising up to 22 repeats in each toxin monomer. However, this resemblance should not be taken to imply a common pore architecture, gating mechanism or evolutionary origin. In TRP channels, an ion-conducting pathway is formed by transmembrane helices within each subunit, whereas an α-LTX pore is generated by receptor-directed insertion following extensive rearrangement of the toxin’s N-terminal regions. The presence of ankyrin-repeat scaffolds in α-LTX, TRPA1 and TRPV1 therefore provides only an intriguing visual and conceptual parallel, but it should not be interpreted as evidence of shared ancestry or convergent evolution of their molecular architectures. The ankyrin repeats are cytoplasmic regulatory domains in TRP channels, whereas the α-LTX ankyrin repeats form part of an extracellular toxin scaffold that supports receptor engagement and pore assembly [32].

Another potential parallel concerns the dynamic permeability reported for TRPA1 and TRPV1. During sustained agonist stimulation, both channels can display time-dependent changes in ion selectivity and increased permeability to large organic cations, a phenomenon commonly described as pore dilation [48,49,50]. The structural interpretation of these observations remains debated, however, and increased dye or organic cation permeability does not necessarily demonstrate simple physical enlargement of the channel pore. It would therefore be premature to equate the proposed dilated states of TRP channels with the much larger α-LTX pore. Nevertheless, the conceptual comparison highlights an important difference in degree and duration (Table 1). TRPA1 and TRPV1 are endogenous channels whose permeability is controlled by reversible gating and cellular regulatory mechanisms. By contrast, receptor-bound α-LTX inserts an exogenous, persistent cation-conducting pathway into the neuronal membrane. In isolated nerve terminals, α-LTX initially stimulates fast vesicular release but can subsequently cause gradual cytoplasmic amino acid efflux, ATP depletion and loss of terminal integrity [51,52].

The functional convergence is especially clear when α-LTX is compared with TRPV1. Capsaicin, noxious heat and extracellular acidification activate the endogenous tetrameric TRPV1 pore, producing cation influx, nociceptor depolarisation and pain [45,47]. α-LTX instead binds neuronal surface receptors and inserts its own tetrameric pore into the membrane. If this occurs in a receptor-expressing nociceptor, the predicted consequences are, similarly, depolarisation, Ca^2+^ entry and release of nociceptive transmitters and neuropeptides. Thus, although the molecular mechanisms are fundamentally different, the two systems can converge on a common physiological sequence: cation influx, sensory neuron excitation and pain.

This comparison becomes even more intriguing in the context of spider venom evolution. Several tarantula toxins have evolved to target vertebrate nociceptive ion channels, particularly TRP and other channels involved in pain sensation [10,53,54]. The double-knot toxin, for example, binds the outer pore of TRPV1 and stabilises the channel in an open state, thereby commandeering an ion-conducting system already present in the nociceptor membrane [10]. These venoms therefore operate primarily by modulating endogenous sensory transducers already present in vertebrate neurons. In contrast, widow spiders appear to have evolved a fundamentally different strategy. Rather than simply targeting ion channels, α-LTX functions as a giant extracellular ankyrin-repeat molecular machine capable of assembling a constitutively permissive cation-permeable pore directly within a neuronal membrane [32].

α-LTX is exceptional among venom neurotoxins. Many toxins that modulate neuronal ion channels are compact, disulfide-stabilised peptides, including conotoxins, inhibitor cystine-knot peptides, three-finger toxins and Kunitz-type toxins. α-LTX is an approximately 130 kDa protein of about 1200 amino acids, with 22 ankyrin repeats accounting for much of its sequence. Rather than functioning as a rigid ligand for an endogenous channel, α-LTX undergoes tetramerisation, receptor-directed membrane insertion and extensive conformational rearrangement to construct its own ion-conducting pathway. It therefore behaves more like a receptor-targeted pore-forming molecular machine than a conventional venom peptide.

Both tarantula and widow spider neurotoxins appear to converge on similar intracellular pathways and sensory outputs. Sustained activation of TRPV1 produces depolarisation, Ca^2+^ entry and downstream signalling involving phospholipase C, thereby promoting neuropeptide release and neurogenic inflammation. α-LTX-induced pore formation can generate the same proximal events—cation influx, depolarisation and Ca^2+^-dependent secretion. ADGRL1 also initiates G-protein-dependent phospholipase C signalling following α-LTX binding, but this pathway should be distinguished from pore formation [55]. Signalling-deficient ADGRL1 and neurexin constructs can still support α-LTX insertion, demonstrating that receptor-mediated signalling is not required to generate a pore [28,34]. The convergence therefore occurs principally at the level of neuronal excitation and intracellular Ca^2+^ signalling, rather than through an identical receptor-coupled pathway.

At an operational level, α-LTX resembles extracellular pore-forming systems more closely than it resembles classical ion channel-modulating toxins. Soluble components of the complement membrane attack complex, for example, assemble and insert into target membranes to produce an aqueous pore [56]. Bacterial pore-forming toxins provide another broad functional analogy [57]. These systems are structurally and evolutionarily unrelated to α-LTX, but they illustrate the same general principle: a soluble protein complex can be converted into a membrane-spanning permeation pathway. α-LTX is distinguished by coupling this capacity specifically to neuronal receptors, thereby directing membrane permeabilisation towards excitable neuronal cells. Provided receptor-positive nociceptors are among its targets, this unusual architecture could enable exceptionally powerful and persistent sensory activation resulting in severe pain.

Together, α-LTX may represent an evolutionary step from modulation of endogenous nociceptive channels toward deployment of an exogenous ion-conducting apparatus. It is possible that the severe muscle spasms observed during latrodectism may represent only one manifestation of a more fundamental membrane-permeabilising strategy optimised for overwhelming sensory activation. Indeed, vertebrate nociceptors may be particularly vulnerable to such permeation dysregulation due to their intrinsic dependence on calcium-permeable TRP signalling systems and amplification pathways associated with inflammatory pain. In this context, α-LTX could be interpreted not merely as a paralytic neurotoxin, but as a toxin that functionally mimics an extreme pathological state of nociceptive membrane permeation. Such a view places α-LTX unexpectedly close to the broader biology of sensory ion channels and highlights a potentially underappreciated convergence between venom evolution and vertebrate pain transduction mechanisms [32,45,46].

## 5. Future Directions

Neurotoxins provide powerful tools for dissecting neuronal mechanisms because they can either activate or silence neuronal populations according to their molecular targets and modes of action [6,10]. α-LTX, the principal vertebrate neurotoxin of widow spider venom, exemplifies an exceptionally potent excitatory toxin [14,58]. Its effects have traditionally been interpreted through neuromuscular junction hyperactivation and the severe muscle spasms characteristic of latrodectism [14,58]. However, the expression of its well-established receptors—ADGRL1/latrophilin-1 and neurexin-1α—in various sensory neurons raises the possibility that α-LTX also contributes directly to pain by acting on nociceptive neurons (Figure 3) [5,16,20,21,38,39,40].

From this perspective, several questions should be addressed:

(1) Which molecularly defined nociceptor subtypes express functional ADGRL1 and toxin-binding neurexin-1α at the cell surface?

(2) Are these receptors, or combinations of them, enriched in nociceptive pathways relative to non-nociceptive sensory, autonomic, motor and central neurons?

(3) Which NRXN1α splice variants are expressed in nociceptors, and what proportion lacks the SS4 insert that inhibits α-LTX binding?

(4) Can α-LTX directly depolarise and activate isolated nociceptor subtypes?

(5) What are the respective contributions of α-LTX pore formation and ADGRL1-mediated signalling?

(6) Which neurotransmitters and neuropeptides are released from sensory neurons following α-LTX exposure?

(7) What proportion of pain in latrodectism results from direct sensory neuron activation rather than secondary muscle contraction, tissue ischaemia or autonomic dysfunction?

(8) What mechanisms underlie headache during latrodectism?

(9) Do additional α-LTX receptors operate in sensory neurons?

(10) Can α-LTX induce degeneration of nociceptor terminals, or even nociceptor death, under conditions of sustained or excessive activation? High-intensity TRPV1 activation by capsaicin and related agonists can produce prolonged nociceptor defunctionalisation and degeneration of nociceptive fibres [59]. It will therefore be important to determine whether α-LTX can induce analogous long-lasting structural or functional changes in nociceptors. If such effects could ultimately be achieved selectively and safely, they might provide a conceptual basis for new approaches for the treatment of chronic pain.

Modern single-cell atlases provide an important starting point for addressing these questions, but transcript detection alone cannot establish toxin susceptibility. Conventional single-cell RNA sequencing may not distinguish neurexin-1α from other NRXN1 transcripts, resolve SS4 usage or demonstrate surface expression of the corresponding protein. Future studies should therefore combine single-cell or single-nucleus transcriptomics with long-read isoform sequencing, spatial mapping, receptor-specific protein detection and direct toxin-binding assays. The candidate α-LTX receptors identified by these approaches should subsequently be validated using complementary structural and functional strategies, including X-ray crystallography or cryo-EM, molecular dynamics simulations, site-directed mutagenesis, and recombinant expression of wild-type and mutant toxins and receptors. Cell-line models could be used to determine whether individual candidate receptors are sufficient to confer α-LTX binding and functional responses, while confocal microscopy and flow cytometry could enable visualisation. Comparisons across human and experimental animal sensory tissues will also be necessary because receptor abundance and alternative splicing may differ between species [18,39,40,41,60].

Functional validation should extend from isolated cells to intact sensory preparations. Electrophysiology and Ca^2+^ imaging in molecularly identified DRG or trigeminal neurons could establish whether α-LTX produces membrane conductance, depolarisation and action potential firing. Compartmentalised cultures and ex vivo skin–nerve preparations could determine whether peripheral endings are directly susceptible. Measurements of CGRP, substance P, glutamate and ATP would define the secretory and non-vesicular components of the response. Ultimately, nociceptor-selective receptor deletion or silencing, combined with simultaneous assessment of pain behaviour and muscle activity, will be needed to separate direct sensory effects from spasm-associated pain in vivo.

Pore-deficient α-LTX mutants may help distinguish receptor-mediated signalling from direct permeabilisation. α-LTXN4C, for example, retains receptor binding and can stimulate transmitter release despite its impaired pore-forming activity [61]. It should not, however, be treated as a “receptor-only” control: recent work indicates that α-LTXN4C can still produce intracellular Ca^2+^ signals and promote extracellular Ca^2+^ entry through endogenous pathways [62]. The profound membrane permeabilisation caused by wild-type toxins can obscure receptor-specific biology, making non-pore-forming or receptor-selective reagents particularly important. Comparisons among wild-type α-LTX, binding-deficient variants, membrane-insertion mutants and receptor-selective constructs will therefore be desirable [63].

Future cell biological studies should also examine the stoichiometry of α-LTX receptor binding, including that of α-LTX mutants. Such studies could help determine whether mechanical deformation or stretching of the ankyrin-repeat shell is associated with the binding of two, three, or four receptor molecules. Further investigation of how the membrane lipid composition influences α-LTX binding to neuronal receptors and subsequent pore insertion into a membrane would also be valuable. The unique nature of the pore-inserting mechanism could also inspire future biotechnological applications, including in non-neuronal cells. In particular, engineered α-LTX derivatives might potentially provide receptor-directed tools for controlled membrane permeabilisation or delivery of ions and small molecules into selected cells. More broadly, understanding how a large soluble protein undergoes a receptor-assisted transition into a membrane-spanning pore may inform the design of synthetic, stimulus- or receptor-dependent nanopores.

The physiological functions of α-LTX receptors must also be considered. ADGRL1 and neurexin-1α are not toxin receptors in an evolutionary sense; they are endogenous organisers of synapse formation, adhesion, signalling and circuit assembly [28]. Pathogenic NRXN1 variants and abnormal isoform expression are associated with several neurodevelopmental and neuropsychiatric disorders [64], while ADGRL1 haploinsufficiency has been linked to a variable neurodevelopmental phenotype in humans and mice [65]. α-LTX and appropriately engineered derivatives may therefore provide tools for investigating the functions of these receptors in sensory circuits.

The persistence of pain and headache after widow spider envenomation raises the additional possibility that α-LTX initiates sensitisation that outlasts the immediate toxin-induced conductance. Sustained Ca^2+^ entry, neuropeptide release, neurogenic inflammation and secondary immune signalling could lower nociceptor thresholds after the initial exposure. The duration of these symptoms is reminiscent of some migraine attacks, but similarity in duration does not yet establish a shared mechanism. Testing the involvement of trigeminal afferents and CGRP signalling, aided by α-LTX humanised antibodies, would provide a more direct basis for evaluating this possible connection [66,67,68].

## 6. Conclusions

This review highlights an underexplored possibility: α-LTX acts not only at motor and central synapses but also directly on the nociceptive system. Its neuronal receptor specificity, receptor-directed pore formation, Ca^2+^ permeability and capacity to stimulate transmitter and neuropeptide release make it highly relevant to sensory neuron biology. Transcriptomic data support the presence of α-LTX receptor transcripts in human and mouse sensory neurons, but do not yet establish the receptor isoform, surface expression or functional toxin sensitivity.

The central hypothesis emerging from this analysis is that the intense pain of latrodectism is a composite phenomenon. Muscle spasm, autonomic dysfunction and tissue-derived signals probably contribute, but activation of receptor-expressing nociceptors would represent a more direct and previously underappreciated mechanism. Establishing this will require isoform-resolved receptor mapping, direct functional analysis of identified nociceptor populations and in vivo separation of sensory from motor effects. Reframing α-LTX as a potential probe of pain circuits therefore provides a testable new perspective on a well-known toxin and may reveal broader principles governing sustained nociceptor excitation, neuropeptide release and pain transmission.

## 7. Methodology

The review focused on the potential relationships among α-LTX, its neuronal receptors, sensory neurons, and nociceptive signalling. Clinical reports and experimental animal studies were considered as a basis for mechanistic interpretation and hypothesis generation. Article selection was therefore hypothesis driven, with particular emphasis on the evidence connecting α-LTX to pain pathways. Studies of neuromuscular junctions and brain-derived preparations were considered where necessary to establish the molecular mechanism of α-LTX, but were not reviewed comprehensively, as these subjects have been extensively covered elsewhere [5]. We conducted a focused literature search using PubMed and Google Scholar, covering publications available from database inception to July 2026. The search primarily included original research articles, supplemented by selected systematic and narrative reviews. The titles and abstracts were screened using the terms “latrotoxin,” “neurexin,” “latrophilin,” “ADGRL1,” and “CIRL,” together with combinations such as “widow spider AND pain” and “latrotoxin AND pain.” Reference lists and subsequent articles citing key publications were also examined to trace the development of major concepts and determine how early observations and hypotheses were subsequently confirmed, refined, or challenged. Articles not published in English and conference abstracts were excluded.

The transcript-level expressions of ADGRL1, NRXN1, TRPV1, and TRPA1 were summarised from processed public dorsal root ganglion transcriptomic datasets in humans and mice. For human DRG, we reanalysed the processed expression matrix and published subtype annotations from A Reference Atlas of the Human Dorsal Root Ganglion, a human DRG single-cell/single-nucleus atlas comprising 22 annotated neuronal subtypes and 53,596 cells or nuclei [18] (Tables S1–S3). For mouse DRG, we reanalysed the processed Usoskin/Linnarsson mouse DRG single-cell dataset using the published Level 3 neuronal class labels, after excluding non-cell wells, yielding 16 sensory neuron classes and 799 cell wells [41] (Tables S4–S6). In both species, cluster identities were taken from the source datasets and were not reclustered in this project. For each annotated subtype or class and each gene, we recalculated the mean expression, median expression, number of cells, and percentage of cells with detectable expression, defining detectable expression as expression greater than 0 in the processed matrix used for that dataset. Dot plots were generated from these recalculated summary tables, with the genes on the x-axis, source-defined neuronal groups on the y-axis, dot size representing the percentage of positive cells, and dot colour representing the mean expression across all cells in the group. ADGRL1 was interpreted as the canonical latrophilin-1 gene, NRXN1 was assessed at the total gene level only and not as isoform-specific evidence, and TRPV1 and TRPA1 were included as nociceptor and chemosensory marker genes. The subtype-resolved transcript abundance represents computational reanalysis of processed public atlases rather than inspection of published figures, and the resulting dot plots should be interpreted as transcript-level summaries rather than direct evidence of protein abundance or function.

## Figures and Tables

**Table 1:** Qualitative comparison of TRPV1, TRPA1 and α-LTX pore properties [32,35,45,46,47,48,49,50].

Property	TRPV1	TRPA1	α-LTX
Channel type	Endogenous gated channel	Endogenous gated channel	Toxin-assembled pore
Ankyrin repeats	~6	~14–18	~22
Pore nature	Regulated	Regulated	Inserted pore
Ion selectivity	Moderate	Moderate	Poor
Ca^2+^ permeability	High	Very high	Massive
Pore dilation	Yes	Strong	Essentially constitutively large
Conductance	~50–100 pS	~80–120 pS	Hundreds of pS-nS
Main function	Heat sensing	Nociception/stress sensing	Exocytosis/spasms/pain

## Data Availability

Data is contained within the article or Supplementary Materials.

## Supplementary Materials

The following supporting information can be downloaded at: https://www.mdpi.com/article/10.3390/toxins18090393/s1, Table S1: Human subtype-level expression summary (human_drg_ADGRL1_NRXN1_TRPV1_TRPA1_summary); Table S2: Human compact review table (human_drg_ADGRL1_NRXN1_TRPV1_TRPA1_review_table); Table S3: Human broad sensory class summary (human_drg_ADGRL1_NRXN1_TRPV1_TRPA1_broad_class_summary); Table S4: Mouse class-level expression summary (mouse_drg_Adgrl1_Nrxn1_Trpv1_Trpa1_summary); Table S5: Mouse compact review table (mouse_drg_Adgrl1_Nrxn1_Trpv1_Trpa1_review_table); Table S6: Mouse broad sensory class summary (mouse_drg_Adgrl1_Nrxn1_Trpv1_Trpa1_broad_class_summary); Table S7: Human–mouse comparison at the broad sensory class level (human_mouse_broad_class_comparison_ADGRL1_NRXN1_TRPV1_TRPA1).

## Abbreviations

The following abbreviations are used in this manuscript:

ADGRL1	Adhesion G protein-coupled receptor latrophilin-1
ATP	Adenosine triphosphate
Aδ	A-delta
CGRP	Calcitonin gene-related peptide
CIRL1	Calcium-independent receptor for latrotoxin-1
CTF	C-terminal fragment
DRG	Dorsal root ganglion
EGF	Epidermal growth factor-like domain
GAIN	GPCR autoproteolysis-inducing domain
GFP	Green fluorescent protein
GPCR	G protein-coupled receptor
GPS	GPCR proteolysis site
HRM	Hormone receptor motif
IB4	Isolectin B4
LNS	Laminin–neurexin–sex hormone-binding globulin domain
LRRTM	Leucine-rich repeat transmembrane protein
α-LTX	Alpha-latrotoxin
α-LTXN4C	α-latrotoxin N4C mutant
NF200	Neurofilament 200
NRXN1	Neurexin 1
NTF	N-terminal fragment
PTPσ	Protein tyrosine phosphatase sigma
RBL	Rhamnose-binding lectin-like domain
RNA	Ribonucleic acid
SS4	Splice site 4
TM	Transmembrane domain
TRP	Transient receptor potential
TRPA1	Transient receptor potential ankyrin 1
TRPV1	Transient receptor potential vanilloid 1
